# Finding an optimal rehabilitation paradigm after stroke: enhancing fiber growth and training of the brain at the right moment

**DOI:** 10.3389/fnhum.2014.00381

**Published:** 2014-06-27

**Authors:** Anna-Sophia Wahl, Martin E. Schwab

**Affiliations:** ^1^Brain Research Institute, University of ZurichZurich, Switzerland; ^2^Department of Health, Sciences and Technology, ETH ZurichZurich, Switzerland

**Keywords:** stroke, rehabilitation, Nogo-A, critical time window, plasticity, training

## Abstract

After stroke the central nervous system reveals a spectrum of intrinsic capacities to react as a highly dynamic system which can change the properties of its circuits, form new contacts, erase others, and remap related cortical and spinal cord regions. This plasticity can lead to a surprising degree of spontaneous recovery. It includes the activation of neuronal molecular mechanisms of growth and of extrinsic growth promoting factors and guidance signals in the tissue. Rehabilitative training and pharmacological interventions may modify and boost these neuronal processes, but almost nothing is known on the optimal timing of the different processes and therapeutic interventions and on their detailed interactions. Finding optimal rehabilitation paradigms requires an optimal orchestration of the internal processes of re-organization and the therapeutic interventions in accordance with defined plastic time windows. In this review we summarize the mechanisms of spontaneous plasticity after stroke and experimental interventions to enhance growth and plasticity, with an emphasis on anti-Nogo-A immunotherapy. We highlight critical time windows of growth and of rehabilitative training and consider different approaches of combinatorial rehabilitative schedules. Finally, we discuss potential future strategies for designing repair and rehabilitation paradigms by introducing a “3 step model”: determination of the metabolic and plastic status of the brain, pharmacological enhancement of its plastic mechanisms, and stabilization of newly formed functional connections by rehabilitative training.

## Introduction

The human brain works wonders to fulfill the requirements of every-day life. These unique capacities are then fully esteemed when all of a sudden even simple activities fail or become a problem: cerebral strokes leave the victims with often large psychical and physical impairments—from vision problems to aphasia and motor deficits—leading to the number one cause of adult disability worldwide with great impact on public health. In the acute phase, “time is brain”—ruptured blood vessels (hemorrhagic stroke) or aggregates of platelets and blood cells that clog cerebral blood vessels (ischemic stroke) cause acute shortage of glucose and oxygen resulting in metabolic distress and long-term neuronal cell loss. The destruction process is complex and can only be dampened in the case of the ischemic stroke by very early intervention (within 4–6 h) with thrombolysis, (Hacke et al., 2008). Currently, only about 10% of all stroke patients reach a hospital early enough or fulfill the criteria for being able to receive thrombolysis in the therapeutic time window. Prognosis and recovery then depend on the location and extent of the stroke lesion. Clinically, the most successful therapy to further enhance this recovery of function is rehabilitative training. Rehabilitation as a term “to reach and maintain optimal functioning in physical, intellectual, psychological and/or social domains” (WHO. International classification of functioning disability Health ICF. Geneva: WHO; 2001) is evidence based medicine and does not exclude a specific subgroup of patients.

Nevertheless, for many rehabilitative interventions, in particular those for long-term or chronic rehabilitation, robust data or adequately controlled studies are lacking (Quinn et al., 2009): e.g., comparisons between different training methods in current use could not show that any particular physiotherapy or stroke rehabilitation strategy is superior to another (Johansson, 2000).

Consequently optimal rehabilitation strategies can only be defined if we understand the way in which training and the rehabilitation protocol influences the neurobiology of the central nervous system with priority on the aspects of timing, kind and intensity of rehabilitative training. Measurable endpoint criteria for rehabilitative outcome are required in order to achieve two purposes: the adjustment of the ideal rehabilitative strategy to the individual patient, and the choice of the optimal therapy protocol.

In this review we focus on mechanisms of spontaneous recovery after stroke, on rehabilitative designs to enhance plasticity, on growth promoting mechanisms with an emphasis on anti-Nogo-A immunotherapy, and on the time windows of rehabilitative training and pharmacological interventions and the combination of both.

## Mechanisms of spontaneous recovery after stroke—from human patients to animal models

For many years people have thought that the hardware of the brain is that “hard”, that once an incident such as stroke happens, brain areas and functions are lost forever. The old paradigm of the adult CNS as a stable and static structure, consisting of billions of nerve cells and circuits, has now been replaced by a much more dynamic view of the CNS which includes processes of growth, connectivity changes and areal remodeling that can occur after CNS injury or stroke and plays an important role in recovery and functional repair.

Spontaneous recovery is seen in stroke patients weeks to months after the incident. However, due to variability across subjects and across neurological domains efforts of summarizing this process with precision have been frustrating. Among the most obvious factors that contribute to the extent of spontaneous recovery are infarct size, infarct location, age and pre-stroke disability (Cramer, 2008). Most spontaneous recovery tends to occur within the first 3 months. While patients with milder deficits achieve spontaneous recovery more quickly than patients with more severe deficits, the pattern of spontaneous recovery can also differ within the same patient for different functions (Cramer, 2008).

### Spontaneous recovery of sensorimotor function in humans

Motor recovery has been among the most often examined because motor impairments belong to the symptoms that are most frequently and precisely diagnosed after stroke (Gresham et al., 1998; Rathore et al., 2002; Langhorne et al., 2009). Motor impairment can be regarded as a loss or limitation of function in muscle control or movement or a limitation in mobility. It is a focus of physiotherapy or occupational therapy in terms of stroke rehabilitation (Langhorne et al., 2009). The natural history of motor recovery is considerably heterogeneous: the first voluntary movements can be seen anywhere from 6 to 33 days after a hemiplegic stroke (Twitchell, 1951). The largest improvement occurs in the first 30 days after stroke, though significant progress is still found in patients with more severe deficits up to 90 days after stroke (Wade, 1983; Duncan et al., 1992, 1994, 2005). Studies on arm disability revealed that a maximum of function is reached by 80% of the patients within 3 weeks and by 95% of patients within 9 weeks (Nakayama et al., 1994). Still significant long-term improvement is found if arm function starts to ameliorate 16 weeks after stroke onset (Broeks et al., 1999).

Insights into the underlying remodeling and re-organization processes for functional recovery in the brain after stroke can be obtained in human patients via functional neuroimaging methods and brain mapping. These data suggest that recovery of motor function after stroke leads to brain-wide modifications in neuronal activity patterns and connectivity (Rehme and Grefkes, 2013). While initially tissue function and neurophysiological responses are diminished within the injured primary neocortex, cortical function increases over time (Marshall et al., 2000; Calautti et al., 2001; Feydy et al., 2002; Grefkes and Fink, 2011). In terms of good functional outcome one of the major correlates is the degree of recovery of neurophysiological activity in the affected primary cortical areas (Cramer, 2008). In other terms: the best behavioral outcomes are associated with the greatest restoration/remodeling of brain function towards the normal state of organization (Ward et al., 2003; Zemke et al., 2003; Ward, 2004; Murphy and Corbett, 2009). This is true even if the post-stroke behavior is far from being identical to the pre-stroke motor kinematics. In particular the extent of corticospinal tract integrity is positively correlated to functional recovery as revealed by transcranial stimulation of the motor cortex (M1) and its efferents after stroke (Talelli et al., 2006). In general, if an ischemic event occurs, those areas are recruited for structural and functional modification which are either close or functionally related and connected or both. Therefore, after a small stroke, peri-infarct tissue is mainly involved that has similar function. By contrast, after a large stroke, tissue that has similar functions might be only found at more distant sites or in unaffected regions of the contralateral hemisphere, where still enough capacity for structural remodeling remains (Murphy and Corbett, 2009).

### The role of the premotor and contralesional motor cortex

Which areas are activated and what they contribute in terms of beneficial re-organization for functional recovery is still under debate: a meta-analysis revealed that activation of premotor areas and the contralesional primary M1 are consistent findings (Rehme et al., 2012; Rehme and Grefkes, 2013). Interactions between premotor areas and the lesioned primary M1 are directly related to recovery and functional outcome. For example, Johansen-Berg et al. (2002) showed that disruption of dorsal premotor cortex activity by transcranial magnetic stimulation (TMS) over both the ipsi- and contralateral hemisphere lead to a deterioration of performance in stroke patients, but not in healthy controls (Johansen-Berg et al., 2002). The exact role of the activation of contralesional M1 is a subject to controversy: longitudinal functional MRI studies revealed enhanced neuronal activity in motor-related areas in both hemispheres after a large stroke. But then during the first 12 months post-stroke this activity returns to unilateral levels similar to those of healthy controls for those patients with good motor recovery (Ward et al., 2003). Remaining increased activity in the contralesional M1 was often associated with poor outcome. Further studies have demonstrated that inhibition of contralesional M1 activity using repetitive TMS may lead to ameliorated motor performance of the stroke-affected hand in the subacute and chronic phase (Nowak et al., 2008; Takeuchi et al., 2012). In contrast, Rehme et al. (2011) found that increases in contralesional M1 activity over the first 10 days after stroke correlate with the amount of spontaneous motor improvement in initially more impaired patients. These data suggest a supportive role for functional recovery in the early phase after stroke for the contralesional M1. In addition, disrupting contralesional M1 activity with TMS resulted in a deterioration of motor-performance of the stroke-affected hand of stroke patients with capsula interna infarcts (Lotze et al., 2006). A clear time-, size or lesion-location- dependent influence of the contralesional M1, be it either beneficial or harmful for functional recovery, remains to be demonstrated.

### Changes in cortical excitability, lateralized activation and somatotopic re-mapping

For the above described remodeling and recruitment of areas three main forms of reorganization have been described: (1) increased cortical excitability in cortical regions distant from, but connected to the stroke core; (2) reduced lateralized activation; and (3) somatotopic modifications within intact cortical regions.

Increased activity, as a first form of reaction to stroke in areas which before stroke formed a distributed network, has been described many times (Brion et al., 1989; Chollet et al., 1991). This phenomenon occurs in several cortical areas which include motor, language, attention and visual functions (Cramer, 2008). Widespread areas of cortical hyperactivity appear days after stroke and diminish within months post incident (Ward, 2004). This form of modification in cortical excitability is thought to be a result of the down-regulation of the *α*1 *γ*-amino butyric acid receptor subunit and a decrease in *γ*-amino butyric acidergic inhibition (Neumann-Haefelin et al., 1998).

The second form of reaction to stroke—reduced lateralized activation—reflects the increased activity in the contralesional hemisphere, which reduces the extent of interhemispheric balance as demonstrated in many stroke studies (Weiller et al., 1993; Seitz et al., 1998). Reduced lateralized activation is a common brain response not only seen in stroke but also in other neurological contexts such as epilepsy, traumatic brain injury and multiple sclerosis (Cramer, 2008). The exact function of this reduced laterality remains to be elucidated: it may be just a subtype of the described increased activity as described in the first form or a passive event reflecting a reduced interhemispheric inhibition resulting from the stroke. Another interpretation is that the contralesional hemisphere has to take over functions that were previously based in the ipsilesional hemisphere.

Both phenomena, increased cortical excitability and reduced laterality, are related to spontaneous functional recovery (Cramer, 2008). Both are time dependent, increasing in the early weeks after stroke and decreasing over months thereafter. This decrease is greater among stroke patients with stronger functional recovery while the persistent increased activity over both hemispheres is greatest in those patients with the poorest outcome (Ward et al., 2003; Cramer and Crafton, 2006). A relation to increased susceptibility for seizures and phantom pain is possible.

The third response to ischemic injury—somatotopic reorganization—implies that intact cortical regions—in particular within the perinfarct area—reassign their functions which they subserved before stroke and take over function, which have been affected or lost by the ischemic event. Some studies suggest that the largest degree of somatotopic reorganization is associated with very large stroke injuries (Cramer and Crafton, 2006). Such map shifts occur in primary and secondary cortical areas (Byrnes et al., 2001).

### Animal models to study stroke induced cortical re-organization on the anatomical and molecular level

As studies in stroke patients have limitations, animal models of stroke have been used to describe remodeling and reorganization processes on the macro and molecular level. Although spontaneous recovery in animals tends to occur earlier (depending on stroke size), imaging and mapping data show a number of analogues between recovery in animals and in humans: connectivity changes between sensorimotor cortex and deep grey matter structures after middle cerebral artery occlusion (MCAO) in rats were comparable to results in human stroke patients (van der Zijden et al., 2007). fMRI studies concentrating on the affected upper limb in rats have described a shift in laterality of activation after stroke such that early after stroke, brain activation during affected paw stimulation is mainly in the contralesional cortex, later after stroke activity shifts toward the normal pattern, that is the ipsilesional cortex (Dijkhuizen et al., 2001, 2003). Hsu and Jones (2006) found that the larger the ischemic insult the stronger the activity in the contralesional M1. In accordance with human studies van Meer et al. (2012) could show that functional recovery after MCAO in rats was correlated with the extent of preservation or restoration of the ipsilesional corticospinal tract in combination with reinstatement of interhemispheric neuronal signal synchronization and normalization of focal network organization.

New mapping methods allow describing somatotopic map shifts in animals in greater detail: a recent study using light based motor mapping in transgenic mice expressing light-sensitive channelrhodopsin-2 before and after focal ischemic lesions of the forelimb sensorimotor areas revealed decreased motor output in the infarcted area and spatial displacement of sensory and motor maps (Harrison et al., 2013). While strokes in sensory cortex caused the sensory map to move into the M1, a stoke in the M1 lead to a compensatory increase in peri-infarct cortical motor output, but did not affect the position or excitability of the sensory maps. *In vivo* 2-photon calcium or voltage sensitive dye imaging furthermore opens up new possibilities to study the reorganization of complex neuronal networks and their functional relevance for stroke recovery (Winship and Murphy, 2008; Stetter et al., 2013). Anatomically, different studies have demonstrated that map-shifts and re-mapping can be accompanied by axonal sprouting (Carmichael, 2003), and dendritic spine turnover (Brown et al., 2008, 2009, 2010). Using different tracing techniques, Starkey et al. (2012b) could show which neurons take over when functional map shifts occur: if the forelimb M1 in rats was destroyed, neurons in the hindlimb area took over to enable functional recovery of the forelimbs. This functional shift was based on sprouting of new axon branches from hindlimb corticospinal fibers into the cervical spinal cord, followed by retraction of the original lumbar projecting axon and thus a conversion of a hindlimb into a forelimb projecting neuron.

Animal studies have also provided first insights on underlying molecular changes. A unilateral infarct is associated with a number of growth related processes, in some cases bilaterally. These events include the induction of inflammatory markers, grow-promoting and inhibiting genes, cell-cycle regulatory genes and genes involved in synaptogenesis, dendritic branching and neuronal sprouting as reviewed elsewhere (Li and Carmichael, 2006; Popa-Wagner et al., 2007).

Three major phases of stroke reaction and repair are often distinguished (Cramer and Crafton, 2006): the first epoch is the acute reaction to the injury and takes place in the initial hours when modifications become apparent in blood flow, edema, metabolism and inflammation. A second epoch is related to repair, starts in the first days post stroke and is on-going for several weeks. During this epoch spontaneous recovery is seen and endogenous repair related events reach their peak levels. The third epoch begins weeks to months after stroke when spontaneous recovery has reached a plateau and represents a stable but still modifiable chronic phase.

On the molecular level stroke induces neuronal growth-promoting genes in sequential waves post insult to initiate axonal sprouting in the peri-infarct cortex, as initially shown in a rat somato-sensory cortex (barrel field) infarct model (Carmichael et al., 2005): in the early phase immediate early genes and growth related mRNAs such as SPRR1 are induced 3–7 days after stroke. Typical growth cone constituents such as GAP43, CAP23 and MARCKS as well as the transcription factor c-Jun are expressed from day 3 onward. Subsequently, the cell adhesion molecule L1, cyclin-dependent kinase inhibitor p21 and embryonic tubulin isoform alpha1 tubulin are induced, followed by the expression of cytoskeletal reorganization genes such as SCG10 and SCLIP. This pattern of growth gene expression described is unique for axonal sprouting as a stroke response compared to expression profiles in neuronal development, peripheral or other CNS injuries (Li et al., 2010). Furthermore, in an early response to stroke (Mattson, 2008; Carmichael, 2012), several neurotrophic factors such as brain-derived neurotrophic factor (BDNF), nerve growth factor (NGF) and neurotrophin 3 (NT-3) as well as fibroblast growth factor (FGF)-2 and insulin-like growth factor (IGF-1), epidermal growth factor (EGF) and glial cell line-derived neurotrophic factor (GDNF) are up-regulated. Each neurotrophic factor species shows a different temporal and cellular distribution pattern (Abe, 2000): while GDNF is mainly expressed by neurons, CNTF induction was predominantly observed in astroglia of the marginal region and VEDF gene expression was found in both non-neuronal and neuronal cell types after stroke.

Axonal sprouting not only requires the induction of growth-promoting programs within perinfarct neurons, but also a reduction in the growth inhibitory environment (Carmichael, 2006): axonal growth inhibition in the adult CNS is mediated through three general classes of proteins: myelin associated proteins (Nogo-A, myelin-associated glycoprotein, oligodendrocyte myelin glycoprotein), extracellular matrix proteins (e.g., chondroitin sulfate proteoglycans) and repulsive cues for growth cones known mainly from development (e.g., ephrins, semaphorins). Interestingly, messenger RNAs for the chondroitine sulfate proteoglycans aggrecan, phosphacan and versican were found to be induced later after stroke than the early and middle phase of the growth-promoting gene expression. A small number of growth inhibitory proteins including Nogo-A (Jiang et al., 2009), ephrin A5, semaphoring IIIa and neuropilin 1 are induced in the early phase, however, but down-regulation of Nogo receptor components were also seen (Li et al., 2010).

Not only a temporal expression pattern of growth promoting and inhibiting genes can be detected, but also the spatial distribution plays a role to induce the brain’s self-repair processes at the right location: axonal sprouting e.g., in the peri-infarct cortex takes place in a distinct environment close to but larger than the glial scar. Thus, within the glial scar representing the wall that separates the stroke core from the surviving per-infarct tissue both, growth-promoting and growth inhibiting factors are induced while the growth-permissive and peri-infact cortex shows a reduction of the levels of growth inhibiting molecules such as chondroitin sulfate proteoglycans. In contrast, neurotrophins such as BDNF are highly up-regulated in the growth-permissive penumbra und repressed in the stroke core (Lanfranconi et al., 2011).

Taken together, the data on the time and space dependent processes of intrinsic repair mechanisms after stroke suggest a critical period or time window, in which the CNS recruits factors for plasticity that enhance functional recovery. One of the most crucial questions that has to be addressed from a clinical perspective is whether this period characterized by map shifts, fiber growth and major functional and structural changes is also the time window in which rehabilitative interventions should be initiated. We now give an overview on rehabilitative and repair strategies with an emphasis on timing, kind and intensity.

## Strategies to enhance plasticity after stroke

### Growth and plasticity enhancing treatments

Since the discovery of nerve growth factors and factors that prevent neuronal outgrowth and survival, it became a goal in experimental animal studies to apply or induce growth-promoting factors and inhibit the inhibiting ones. Several preclinical studies have examined various growth factors, hormones and cytokines with the aim to enhance motor rehabilitation—including prominent candidates such as NGF, glia (GDNF) and BDNF, IGF, erythropoietin and the granulocyte colony-stimulating factor. All have met with variable levels of success in animal models; some initial clinical studies have started (The BDNF study group (Phase III), 1999; Nagahara and Tuszynski, 2011).

In adult rats with large strokes, the administration of BDNF resulted in improved recovery rates (Schäbitz et al., 2004), while the beneficial effect of rehabilitation on the improvement of forelimb function was prevented in animals treated with a BDNF antisense oligonucleotide (Ploughman et al., 2009). The translation of these results into clinical trials remains challenging and is a matter of safety concerns: in the case of BDNF applied as a neuro-protective agent after stroke, the administration of very large quantities would be necessary as well as repeated dosing to overcome the limited amount of protein that reaches the CNS, even with transient disruption of the blood-brain barrier after stroke. The adverse effects of these high dosages have not been extensively studied in animal models (Nagahara and Tuszynski, 2011). Furthermore, the largest clinical trial of erythropoietin therapy revealed that, compared with placebo, erythropoietin administration was associated with an increased risk of mortality in patients with acute stroke (Ehrenreich et al., 2009).

Other experimental approaches to enhance the intrinsic regeneration ability of CNS axons include injecting cAMP analogs to influence intracellular signaling pathways (Hannila and Filbin, 2008), knock down of the protein synthesis inhibitor PTEN (Liu et al., 2010) or blocking the small GTPase RhoA (Ellezam et al., 2002).

Promising results have also been gained if inhibition of neuronal plasticity and outgrowth was decreased either by: (1) digesting growth restricting ECM proteoglycans with enzymes such as chondroitinase ABC; (2) by blocking the growth inhibitory protein Nogo-A; or (3) by grafting growth permissive cells.

The bacterial enzyme chondroitinase ABC digests the glycosaminoglycan chains of the chondroitin sulfate proteoglycans (CSPGs) which are part of the extracellular matrix and usually up-regulated in astrocyctes and oligodendrocytes after CNS injury (García-Alías and Fawcett, 2012). Chondroitinase ABC treatment reduces scar formation and enhances axonal regeneration and sprouting as first shown in several studies after experimental spinal cord injury (Moon et al., 2001; Bradbury et al., 2002; Huang et al., 2006). After stroke, chondroitinase ABC administration promoted functional recovery (Hill et al., 2012; Starkey et al., 2012a). Furthermore, Soleman et al. (2012) could demonstrate that delayed chondroitinase ABC microinjections into the cervical spinal cord induce localized plasticity of the forelimb sensorimotor spinal circuitry without effects on the cortical peri-infarct region.

#### Inhibition of Nogo-A signaling in animal models of stroke

The well-studied protein Nogo-A, a transmembrane protein of about 1200 amino acids including a C-terminal 200 amino acid reticulon (RTN) domain, is involved in several cellular and molecular events contributing to the failure of CNS axons to sprout and reconnect after CNS injury. Function-blocking antibodies against Nogo-A, Nogo receptor (NgR1)-blocking peptides, antibodies against the Nogo receptor subunit Lingo-1, or pharmacological blockade of the signal transducer RhoA and ROCK have been administered in various laboratories in different stroke and spinal cord injury models in rodents and primates (Pernet and Schwab, 2012 for review). Enhancement of behavioral recovery in a variety of sensory-motor tasks as well as anatomical evidence of fiber growth, increased plasticity and re-organization within the cortex, brain stem and spinal cord have been reported (Zörner and Schwab, 2010 for review). Despite different approaches to interrupt Nogo-A signaling, a high degree of similarity in terms of functional recovery and hardware changes in the CNS was found among research groups and injury models. Acute intrathecal anti-Nogo-A antibody infusion over 2 weeks after stroke, with an application starting early after incident (Wiessner et al., 2003; Tsai et al., 2007), or delayed application starting 9 weeks after stroke in adult rats (Tsai et al., 2011) significantly improved forelimb function and was correlated with a significant increase of midline crossing corticospinal fibers originating in the unlesioned sensorimotor cortex. Robust sprouting of new projections from contralesional brain regions into subcortical structures as well as functional reorganization of contralateral sensorimotor areas were reported after anti-Nogo-A immunotherapy in rats (Markus et al., 2005; Cheatwood et al., 2008). Those newly sprouting cortico-efferent axons terminated in the red nucleus, pontine nuclei and spinal cord. A similar effect was found by down-regulation of the Nogo receptor NgR using adenovirus-mediated RNA interference (Wang et al., 2010) or NgR or Nogo-A/B knockout mice (Lee et al., 2004). Anti-Nogo-A immunotherapy was also associated with increases in dendritic length, complexity, and spine density, both in the lesioned and contralesional hemisphere (Papadopoulos et al., 2006). Functional MR-imaging 8 weeks after unilateral MCAO revealed adaptations in the somatosensory system of rats in the anti-Nogo-A antibody treatment group (Markus et al., 2005). Nevertheless anti-Nogo-A immunotherapy is not neuroprotective in the sense that it would reduce stroke lesion size as reported for anti-MAG immunotherapy (Irving et al., 2005). This opens the therapeutic window for anti-Nogo-A immunotherapy in the subacute and even chronic phase.

The described *in vivo* experiments represent essential preclinical tests to validate the efficiency and safety of intrathecal Nogo-A antibody administration. Three different anti-Nogo-A antibodies (IN-1, 11C7, 7B12) have proved efficient in enhancing axonal regeneration and outgrowth both *in vitro* and *in vivo*. In collaboration with Novartis Pharma, a human anti-human Nogo-A antibody has been developed and tested in extensive toxicological studies with intrathecal antibody application in rodents and primates. In a Phase I clinical trial^1^ with 52 acutely injured para- and tetraplegic patients in Europe (European Multicenter Study about Spinal Cord Injury, EMSCI^2^) and Canada pharmacokinetics, safety, tolerance and dosing of intrathecal delivery of the antibody were investigated. The tolerance has been excellent without any adverse effects ascribed to the anti-Nogo-A antibody (Abel et al., 2011). A placebo-controlled Phase II clinical trial is currently in preparation. Anti-Nogo antibodies are also in clinical trials or in preparation for clinical trials for other neurological indications such as multiple sclerosis and amytrophic lateral sclerosis (ALS). For ALS GlaxoSmithKline (GSK) has also developed a humanized anti-Nogo-A antibody (GSK1223249). In a Phase I clinical trial, the intravenous injections of GSK1223249 were well tolerated by the 76 patients enrolled in the study (Pradat et al., 2011).

Several additional molecules restricting axonal growth *in vitro* have been identified including ephrins, netrins, semaphorins and oligodendrocyte myelin glycoprotein (OMgp; Schwab, 1990, 2010; Schwab et al., 1993). Their role *in vivo* after stroke has to be evaluated. How much growth and plasticity of the adult, stroke-injured CNS can be enhanced by single or combined manipulations of growth promoting or inhibitory mechanisms, and if there is a danger of chaotic growth and formation of wrong connections is currently unknown.

Finally, grafting growth permissive cells, such as bone-marrow mesenchymal cells, cord blood cells, fetal cells and embryonic cells as a form of restorative therapy have been studied in animals (Chopp and Li, 2002). E.g., cultivated bone-marrow stromal cells from donor rats were stereotactically implanted into the peri-infarct area in rats resulting in significant recovery of somatosensory behavior. In a first small study, 5/30 stroke patients who received autologous bone-marrow mesenchymal cell transplantation showed beneficial effects in clinical stroke scores (Bang et al., 2005). Such cell-based therapies could influence endogenous neurogenesis, axonal sprouting and synaptogenesis in ischemic brain tissue (Zhang and Chopp, 2009), although their effects may be primarily immune-modulatory or neurotrophic. More detailed and systematic studies are certainly needed.

### Rehabilitative training in clinical and experimental studies

The brain, including the motor system, learns by repetition and training. Many basic mechanisms, however, are still poorly understood, and rehabilitative training is largely evidence-based medicine (European Stroke Organisation (ESO) Executive Committee; ESO Writing Committee, 2008). Nevertheless there are no generally accepted guidelines and no definite recommendations concerning the timing, kind and intensity of rehabilitative training. Clear end point data and randomized controlled clinical trials are often lacking. Furthermore, stroke recovery is a complex process that probably occurs through a combination of restoration, substitution and compensation of functions. For this reason it has been also difficult to translate results from rehabilitative studies in animals to recommendations for rehabilitative schedules in human stroke patients. A majority of clinical studies has been conducted in chronic stroke patients (> 6 months after the stroke) as recruitment of these patients was easier and baseline performance had stabilized (Krakauer et al., 2012). These circumstances lead to functional outcome measurements probably gained largely from compensatory techniques to improve skills for daily living. In contrast, animal studies had a stronger focus on enhancing impairment with more or less detailed analysis how much of the functional recovery was restoration of baseline (motor) function or compensation. Furthermore, the time courses of motor recovery differ among animal and human studies: While recovery in rodent models reaches its maximum around 4 weeks after stroke, human stroke survivors complete most of their recovery within 3 months (Dimyan and Cohen, 2011; Krakauer et al., 2012).

#### Early vs. delayed training

A consensus exists that the effects of early training, whereby “early” should be starting at 1–2 weeks in animals, not earlier (see below), exceed effects of delayed training in terms of functional recovery in both, animals and humans (Nudo, 2006; Murphy and Corbett, 2009; Langhorne et al., 2010; Krakauer et al., 2012). In animal studies, behavioral training after ischemic injury is most effective for restoring behavioral performance, peri-infarct neurophysiological maps and enhanced neuroanatomical changes in the ipsi- and contralesional hemisphere when introduced within the first week of injury (Nudo, 2006). In a rat MCAO stroke model it was demonstrated that functional outcome and dendritic branching patterns in the contralesional hemisphere were restricted when rehabilitative training was initiated 14 and 30 days post insult (Biernaskie et al., 2004). In another study by Hsu and Jones (2005), rats were trained in a skilled forelimb reaching tasking starting 4 or 25 days post stroke. Reaching performance was significantly enhanced in the early trained group. In a small ischemic insult in M1 in squirrel monkeys delayed training resulted in a large decrease in spared hand representation during the spontaneous recovery period that persisted following the delayed training (Barbay et al., 2006).

Concerns about initiating therapy too early following stroke arose from studies where lesion size and cell death rate were seen to be exaggerated after early excessive use of the impaired forelimb in rats while the unimpaired forelimb was casted (Kozlowski et al., 1996). One cause for increased lesion size following early excessive limb training might be NMDA-mediated excitotoxicity in the already hyperexcitable peri-infarct region (Humm et al., 1999). In closer resemblance to clinical practice were animal studies, where training or enriched rehabilitation was initiated a few days after stroke. In these cases early intervention (1–3 days post stroke) again was associated with increased cell-death but also with much improved motor performance on the long-term (Risedal et al., 1999; Farrell et al., 2001). Here, neuronal cell death may be part of a pruning effect in which non- or dysfunctional neurons are eliminated early due to a use-dependent selection. In summary, the overall consensus from animal data is that initiating rehabilitative training 5 or more days after stroke is mostly beneficial and has no adverse effects (Krakauer et al., 2012).

#### Constraint-induced movement therapy (CIMT), robot assisted training and electrical devices to stimulate the rehabilitation process

For human stroke patients two advanced rehabilitative approaches have proven beneficial for functional outcome: constraint-induced movement therapy (CIMT) and robot-assisted training for upper limb function (Langhorne et al., 2009; Liao et al., 2012; Mehrholz et al., 2012). Extensive preclinical studies in rodents and primates have preceded both rehabilitative strategies (Taub et al., 2002). When somatic sensation is surgically abolished from a single forelimb in a monkey, the animal avoids the usage of this forelimb in the free situation, but monkeys can be induced to use the de-afferented extremity by restricting movement of the intact limb continuously for a period of days. This concept was successfully brought into the clinics when chronic stroke patients wore a sling or cast on their less affected arm during 90% of their waking hours for 14 days (Taub et al., 1993). These patients showed a significant increase in the skill and quality of movement as measured by two laboratory tests and a much larger increase in real-world arm use over the period of these 2 weeks than the unrestricted control group. Two studies addressed the question of intensity and timing for CIMT: In the VECTORS study (Dromerick et al., 2009), 52 stroke patients were randomized at about 10 days post stroke to two levels of intensity of CIMT or standard upper extremity therapy. Intense meant 3 h of CIMT vs. 2 h of shaping therapy. After 90 days the motor outcome was worse for the more intensive CIMT group, although there had been no difference at 30 days. This result reflects the fact that too intensive CIMT can turn into an adverse situation for both the patient and the therapist. In the much larger EXCITE study (Wolf et al., 2006) patients started CIMT therapy 3–9 months post stroke and showed greater motor recovery than the usual care group. In addition Lang et al. (2013) revealed that improvements in existing motor abilities were possible with both early (3–9 months post stroke) and delayed (15–21 months post stroke) application of CIMT. However, significant reacquisition of the ability to complete tasks was only detected with early CIMT treatment.

A number of arm and also hand training robots have been developed recently with the aim to allow very intense training without continuous, costly physiotherapy assistance. In the most modern set-ups, training devices are combined with interactive video games that can boost the motivation of the patient for the training and facility e.g., precision movements (e.g., grasping eggs and putting them into a basket). The number of well controlled and standardized outcome studies is still very limited. However, differences are discriminated between recovery of specific movements under “laboratory conditions” and functional gains for daily life activities (Mehrholz et al., 2012). Such studies are needed to exactly know the specific advantages (and potential drawbacks) of robot assisted rehabilitation in stroke (Aisen et al., 1997; Balasubramanian et al., 2010; Mehrholz et al., 2012).

Therapeutic approaches which directly stimulate the PNS or CNS electrically or by magnetic pulses may enhance neuroplasticity during poststroke rehabilitation (Dimyan and Cohen, 2011). Numerous research groups have examined the stimulation of the CNS, specifically the primary M1, by noninvasive approaches such as TMS and direct current stimulation as well as experimentally in animals by the implantation of electrodes. Several studies showed that an increase of the excitability in the stroke-affected ipsilesional M1 by electrical devices resulted in improved motor outcome (Hummel et al., 2005; Malcolm et al., 2007; Ameli et al., 2009; Koganemaru et al., 2010). The mechanisms of action of these techniques are under investigation but might involve changes in synaptic activity, gene expression and increases in neurotransmitter, receptor and neurotrophin levels (Dimyan and Cohen, 2011) or even enhanced fiber sprouting (Martin, 2012). Understanding these mechanisms may provide the basis for novel approaches using closed-loop brain machine interfaces (BMIs) that define optimal stimulation parameters from a priori developed experimental models and correctly modulate ionic currents and extracellular electric fields to provoke and guide plastic changes of the CNS (Gonzalez Andino et al., 2011).

## Combination of different repair and rehabilitation strategies

To maximize the effectiveness of rehabilitative therapies after stroke, it is critical to define when the brain is most responsive to sensorimotor input or extrinsic application of plasticity promoting reagents. This becomes particularly important if different rehabilitative approaches are combined.

In one of the first proof of concept studies for a critical period of heightened neuroplasticity, stroke rats were exposed to an enriched environment in combination with daily sessions of grasping training. The most significant gains in the recovery of forelimb reaching ability were achieved when rehabilitation was initiated early, i.e., 5 days after stroke as compared to 14 and 30 days after stroke. Recovery was associated with increased dendritic branching of layer V M1 neurons in the unlesioned hemisphere—a response that was not detected when rehabilitation was delayed by 30 days (Biernaskie et al., 2004).

A few recent studies in which regenerative therapies and rehabilitation have been combined have been conducted since then. These experiments suggest that designing the combination and their temporal pattern of administration are not going to be trivial (García-Alías and Fawcett, 2012; Starkey and Schwab, 2012). The different experiments have revealed a beneficial combinatorial effect, a detrimental effect, no effect at all, or an effect that depends on the relative timing of plasticity treatment and rehabilitation.

Beneficial effects were described in spinal cord injury rat models when agents against inhibitory molecules in the CNS were combined with growth promoting reagents: García-Alías et al. (2011) reported that the combination of Chondroitinase ABC with neurotrophin NT-3 and an increased expression of the NR2D subunit of the NMDA receptor resulted in better body stability and interlimb coordination compared with the single treatment groups. The behavioral data were correlated with the highest number of sprouting axons in the spinal cord and multisynaptic responses in the motor-neurons. Similar results could be found if anti-Nogo-A antibodies were combined with NT-3 and the NMDA-NR2D subunit (Schnell et al., 2011). Furthermore, the combinatorial treatment of acutely applied anti-Nogo-A antibody followed by delayed Chondroitinase ABC treatment starting 3 weeks after spinal cord injury, and forelimb grasp training starting at 4 weeks was much more effective in terms of functional recovery, sprouting and axonal regeneration than the single treatments (Rehme et al., 2011). In rats with large cortical strokes, inosine, a substance which was shown to improve fine motor control after stroke (Zai et al., 2009), augmented the effects of the Nogo receptor blocker NEP1-40 in the restoration of skilled reaching abilities in rats. Similar functional improvements were seen when inosine was combined with environmental enrichment (Zai et al., 2011).

Several recent experiments—mainly in spinal cord injury—have combined growth-promoting agents with rehabilitative training with somewhat different results: García-Alías et al. (2009) investigated whether chondroitinase-induced plasticity combined with physical rehabilitation promotes recovery of manual dexterity in rats with cervical spinal cord injury. While CSPG digestion combined with forelimb-specific rehabilitation lead to improved manual dexterity, animals treated with chondroitinase ABC in combination with environmental enrichment improved in ladder walking but performed much worse in skilled forelimb tasks than untreated control animals. In a second investigation by Maier et al. (2009) adult rats with large but incomplete cervical spinal cord injury received anti-Nogo A antibodies and simultaneous daily forced treadmill training. The simultaneous rehabilitative therapy clearly worsened the functional outcome compared with either treatment alone. When the forced treadmill training was delayed, however, for 2 weeks after the end of the antibody treatment a very good functional outcome was obtained (Marsh et al., 2011). In contrast to these results in spinal cord injured rats, combination of Nogo receptor blockade with skilled forelimb training in stroke lead to a greater degree of recovery than when either of the treatments were applied alone (Fang et al., 2010).

No additive or adverse effects were reported by Boyce et al. (2007) when neurotrophins were combined with rehabilitative training in spinal cord injured cats. Administration of pharmacological neuromodulators such as amphetamine and cholinergic agonists in combination with rehabilitative training are a matter of debate: early animal research had suggested a beneficial effect of amphetamine in recovery of motor function after stroke which could not be sufficiently reproduced in recent human and animal studies (Krakauer et al., 2012). Only for the anti-depressant fluoxetine, a serotonin-selective reuptake inhibitor, which was applied from 9 days post stroke to 3 months in a human stroke study, an impressive degree of increased motor recovery was found when combined with rehabilitative training (Chollet et al., 2011). For all these studies and their quite diverse outcomes, better knowledge of the neurobiological phenomena and mechanisms triggered by the injury, the spontaneous reaction of the nervous tissue to it, and by the different pharmacological and behavioral interventions is urgently required.

## Future directions for designing optimal rehabilitation schedules

**Figure 1:**
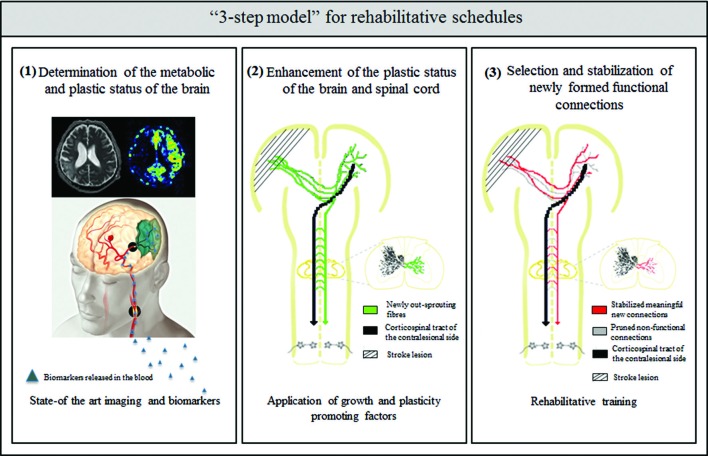
**Schematic overview of the “3 step model”—as a possible roadmap for designing future rehabilitation schedules: (1)** determination of the metabolic and plastic status of the brain by using state- of the art imaging technologies (image taken by the Akashi Municipal Hospital, Japan) and biomarker profiles in the blood and CSF; **(2)** enhancement of intrinsic repair and plasticity mechanisms in the ispi- and contralesional hemisphere as well as the spinal cord by application of growth and plasticity-promoting factors such as anti-Nogo-A antibody or Chondroitinase ABC; and **(3)** selection and stabilization of newly formed functional connections and pruning of non-functional ones by rehabilitative training.

How can we better understand the neurobiology of rehabilitation? What can we learn from the above mentioned animal and clinical studies to improve current rehabilitation schedules for the best possible recovery after stroke? The presence of critical time windows for the application of growth and plasticity promoting agents and of training-dependent plasticity suggests that careful consideration of rehabilitation onset times, tailored training to the type and extent of stroke and the patient’s history are required. Potential future rehabilitation schedules after stroke may therefore include the following “3 step model” (Figure 1):

1. Determination of the metabolic and plastic status of the brain by using state-of-the-art imaging technologies and metabolic markers
2. Enhancement of the plastic status of the brain by the application of growth and plasticity-promoting factors
3. Selection and stabilization of newly formed functional connections by rehabilitative training

One obstacle of the implementation of the optimal restorative therapies is the heterogeneity of stroke as injury location and size differ widely from one patient to another. The ability to assign the right therapy to the right patient would maximize treatment effects. Although clinical scores and a number of imaging methods exist for evaluating the state of the central nervous system and its function after stroke as reviewed elsewhere (Burke and Cramer, 2013), these approaches are often insensitive, cost intensive and have logistical difficulties. Nevertheless, neuroimaging is not only essential for the establishment of acute stroke diagnosis but can also serve as a powerful tool for the characterization of disease progression and monitoring of the response to rehabilitative interventions. Diffusion-weighted imaging (DWI) and perfusion-weighted MRI (PWI) are widely available MRI modalities that provide valuable information about the tissue characteristics of the ischemic core but also of the tissue at risk in the penumbra (Merino and Warach, 2010; Fisher and Bastan, 2012). Further work is needed to optimize the characterization of penumbra imaging for patient triage into adjusted treatment groups. In the near future we expect to learn if penumbra imaging or other early imaging features provide predictive value of critical time windows in which therapeutic interventions should be initiated or maintained and allow stratification of patients into groups for specific types of therapies.

Biomarker profiles in blood and cerebrospinal fluid (CSF) samples could bring a tremendous advance and are currently a focus of genomic and proteomic profiling studies and of systems biology in several laboratories (Stuart et al., 2010; Hemphill et al., 2011; Whiteley et al., 2012). In this regard, a biomarker or a specific combination and profile of biomarkers may not only speed up diagnosis and initiation of acute stroke treatment but may also help to classify and categorize patient groups for prediction of outcome and target the right rehabilitative approach to those stroke patients who would benefit the most.

Why do we suggest a temporal sequence of first enhancing the plastic state by growth promoting agents followed by a phase of rehabilitative training in our “3 step model”?

The current data suggest that the CNS reacts to the injury by an activation of growth and plasticity mechanisms which, however, seem to also represent a vulnerable phase in which forced activity can be harmful: this phase includes a period of GABA-mediated tonic inhibition, which may also be necessary in the first days after the stroke to limit an expansion of the infarct size (Clarkson et al., 2010), as well as homeostatic plasticity mechanisms, which ensure that neurons receive an balanced amount of synaptic input (Murphy and Corbett, 2009). Intrinsic growth and plasticity as well as exogenous enhancement of growth will lead to the formation of a large number of new connections within and between different areas of the injured CNS. In analogy to the situation in early postnatal development, many of these connections may be weak and imprecise. The functionally meaningful ones will now have to be selected and stabilized, while the malfunctional ones should be pruned, in the next, activity-dependent phase of the recovery process.

In the last step of recovery that is based mainly on rehabilitative training the spared and the new circuitry of the CNS is shaped by selection and stabilization of functional connections and pruning of the non-functional ones. Hebbian learning rules might play a crucial role in this step in the sense that Hebbian plasticity mechanisms redistribute synaptic strength to favor the wiring of pathways that are coincidently active (Murphy and Corbett, 2009). Motor learning in development is a very protracted process, requiring huge numbers of repetitions over a period of many weeks and months. Much too less is known today on the optimal time and intensity requirements for rehabilitation learning. To distinguish optimal rehabilitation schedules from less beneficial ones, strict criteria for functional outcome have to be defined that discriminate compensation and substitution from real restoration of previously impaired function. Much remains to be learned and applied in this fascinating and medically most important field of stroke rehabilitation at the interface between basic neuroscience and clinical neurology.

## Conflict of interest statement

The authors declare that the research was conducted in the absence of any commercial or financial relationships that could be construed as a potential conflict of interest.

## Footnotes

^1^http://clinicaltrials.gov/ct2/show/NCT00406016

^2^www.emsci.org
